# Effect of a Plateau Environment on the Oxidation State of the Heart and Liver through AMPK/p38 MAPK/Nrf2-ARE Signaling Pathways in Tibetan and DLY Pigs

**DOI:** 10.3390/ani12091219

**Published:** 2022-05-09

**Authors:** Hong Hu, Yongxiang Li, Yuting Yang, Kexing Xu, Lijie Yang, Shiyan Qiao, Hongbin Pan

**Affiliations:** 1 Yunnan Provincial Key Laboratory of Animal Nutrition and Feed Science, Faculty of Animal Science and Technology, Yunnan Agricultural University, Kunming 650201, China; haiyanghh@163.com (H.H.); yongxiang9831@163.com (Y.L.); 2 State Key Laboratory of Animal Nutrition, College of Animal Science and Technology, China Agricultural University, Beijing 100193, China; yuting_yang1001@163.com (Y.Y.); yang.superman@163.com (L.Y.); 3 College of Animal Science, Anhui Science and Technology University, Chuzhou 233100, China; xkx13635674098@163.com

**Keywords:** Tibetan pig, DLY pig, plateau environment, AMPK, p38 MAPK, Nrf2-ARE, oxidative stress

## Abstract

**Simple Summary:**

Plateau stress is a main factor limiting pig production. There are great differences in oxidation state and antioxidant defense between Tibetan pigs (TPs) and DLY pigs exposed to a plateau environment. However, there are limited reports on the mechanism of adaptation of TPs and DLY pigs to the plateau environment involving a signal pathway related to oxidation state. In the present study, we found that TPs exhibit a stronger heart and liver antioxidant stress capacity than DLY pigs through AMPK/p38 MAPK/Nrf2-ARE signaling pathways under a plateau hypoxic environment.

**Abstract:**

This study evaluated the effect of a plateau environment on the heart and liver oxidation state of Tibetan pigs (TPs) and DLY pigs through analyzing AMPK, p38 MAPK, and Nrf2 signaling pathways. Twelve 120-day-old TPs and twelve 120-day-old DLY pigs were randomly divided into two groups in a plateau environment for three weeks. Exposed to a plateau environment, TPs exhibited a lower (*p* < 0.05) malondialdehyde level but higher (*p* < 0.05) glutathione, superoxide dismutase (SOD), glutathione peroxidase (GSH-Px), and total antioxidant capacity (T-AOC) activities in the liver and heart than those observed in DLY pigs. TPs also showed higher (*p* < 0.05) mRNA levels of SOD and GSH-Px in the liver and heart compared with those of DLY pigs. The TPs showed higher (*p* < 0.05) mRNA and protein levels of AMPK and Nrf2 in the liver and heart compared with those of DLY pigs. Furthermore, TPs showed higher (*p* < 0.05) mRNA and protein levels of p38 MAPK in the heart and higher mRNA levels of p38 MAPK in the liver compared with those of DLY pigs under a plateau environment. In summary, TPs possess a stronger antioxidant capacity in the heart and liver than that of DLY pigs in a plateau environment through AMPK/p38 MAPK/Nrf2-ARE signaling pathways.

## 1. Introduction

The development of modern animal husbandry shows great harm in the commercial production of pigs, especially the large-scale intensive feeding mode in which oxidative stress negatively affects tissues and organ function [1,2]. In order to meet the demand for animal protein of highland humans, the various western lean pig breeds were raised in plateau environments. However, the plateau altitude is more than 500 m above sea level with characteristics of low temperature, low pressure, low oxygen partial pressure, and strong radiation causing oxidative stress in pig production [3,4,5]. Acute hypoxia (low blood oxygen) may occur in pigs upon entering a plateau. Antioxidant enzyme activity decreases during tissue hypoxia or ischemia in animals and humans, while oxidative stress biomarkers, oxygen free radicals, and lipid peroxidation products increase in respiratory, blood, urine, and tissue samples [6,7].

The Tibetan pig (TP) is a typical type of plateau miniature pig, which has lived in the low oxygen areas of a high-altitude environment for many years [8,9,10]. The tissues and organs of TPs gradually developed strong anti-stress adaptations, well-suited for the characteristics of a plateau environment. Meanwhile, the Duroc × (Landrace × Yorkshire) (DLY) hybrid pig is a lean breed with fast growth rates and high feed conversion efficiency [11,12]. Generally, western lean pig breeds including Large White, Duroc, and Landrace are prone to stress in a poor environment compared with that of TPs [8,13,14]. However, the molecular mechanism of adaptation of TPs to a plateau environment does not adequately describe the signaling pathway involved.

The low oxygen partial pressure on a plateau environment readily causes hypoxia in animals. Hypoxic damage is one of the main underlying causes of stress in the heart and liver [15,16]; however, only a few reports describe its specific mechanism. In this study, differences in oxidative stress and antioxidant defense responses in the heart and liver of TPs and DLY pigs were explored in a plateau environment. The mechanism of high-altitude tolerance in TPs was clarified by focusing on the relationship between oxidation state and adenosine 5′-monophosphate-activated protein kinase (AMPK), p38 mitogen-activated protein kinase (p38 MAPK), and nuclear factor erythroid 2-related factor 2 (Nrf2) signaling pathways.

## 2. Materials and Methods

### 2.1. Pig and Experimental Design

The experiment was approved by the Animal Care and Ethics Committee of Yunnan Agricultural University, China (no. YNAU20201304). Twelve 120-day-old TPs and twelve 120-day-old DLY pigs, obtained from the cooperative farm of Yunnan Agricultural University, were randomly divided into two groups (TP and DLY groups). The experiment was conducted at the farm of Yunnan Agricultural University (Kunming, Yunnan-Kweichow Plateau, China) at an elevation > 1900 m above sea level. The basal diet was formulated according to the feeding standard of swine (NY-T 65-2004, Table 1) [17]. TPs and DLY pigs were given free access to water and feed, and the experiment lasted for 3 weeks.

### 2.2. Sample Collection

At the end of experiment, the 12 pigs from each group were slaughtered for sampling after fasting overnight. Heart and liver samples were collected and preserved at 80 °C for analysis of the oxidation index, protein level, and mRNA level.

### 2.3. Analysis of the Oxidation Index

Malondialdehyde (MDA), total antioxidant capacity (T-AOC), superoxide dismutase (SOD), glutathione (GSH), catalase (CAT), and glutathione peroxidase (GSH-Px) in the liver and heart were determined using commercial assay kits according to the manufacturer’s instructions (Jiancheng Bioengineering Research Institute, Nanjing, China).

### 2.4. Analysis of Nrf2, AMPK and p38 MAPK Levels

Porcine Nrf2, AMPK, and p38 MAPK ELISA assay kits (Jiancheng Bioengineering Research Institute, Nanjing, China) were used to measure the protein levels of Nrf2, AMPK, and p38 MAPK, respectively, in the liver and heart. Porcine Nrf2, AMPK, and p38 MAPK were measured at 450 nm using the Multiskan SkyHigh Microplate Spectrophotometer (Thermo Scientific, Waltham, MA, USA), and its absorbance was negatively correlated with antigen density of liver and heart samples.

### 2.5. Gene Expression Analysis

Total RNA was extracted from the liver and heart using E.Z.N.A. total RNA kit I (Omega BIO-TEK, Norcross, GA, USA). The genomic DNA was removed using DNase (Omega Bio-Tek, USA). SOD, GSH-Px, CAT, Nrf2, AMPK, and p38 MAPK mRNA expression levels were detected using quantitative real-time polymerase chain reaction (qRT-PCR) with the primers shown in Table 2. The qRT-PCR was conducted using TB green Premix Ex Taq (Takara, Shiga, Japan) according to the manufacturer’s instructions, and the following cycling conditions were employed: 95 °C for 30 s, 40 cycles of 95 °C for 5 s, and 60 °C for 30 s [18]. The data were normalized using the Ct of β-actin and the mRNA level of target gene was calculated using the 2^−ΔΔCT^ method [19].

### 2.6. Statistical Analysis

Data were analyzed using Student’s *t*-test to compare the TP and DLY groups using SPSS 18.0. Values were presented as means ± SEM, and *p* < 0.05 was considered statistically significant.

## 3. Results

### 3.1. Effects of the Plateau Environment on MDA in TPs and DLY Pigs

**Figure 1:**
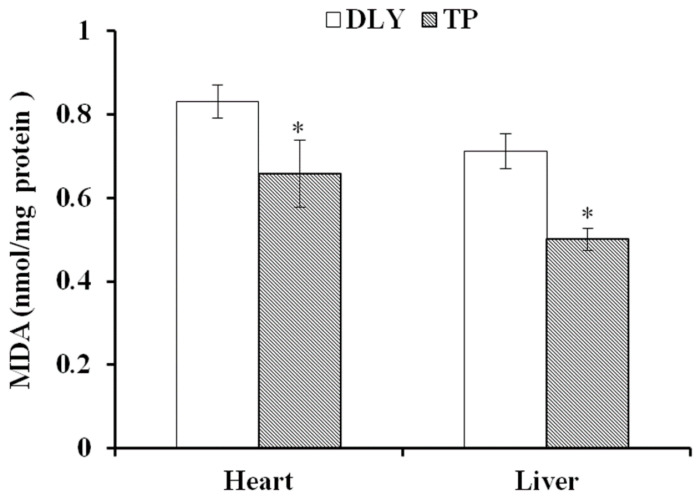
Effects of the plateau environment on MDA in heart and liver of TPs and DLY pigs (*n* = 12). * Significant difference (*p* < 0.05). MDA = malondialdehyde.

The effects of the plateau environment on the MDA of TPs and DLY pigs are shown in Figure 1. Exposed to the plateau environment, TPs exhibited lower (*p* < 0.05) MDA levels in the heart and liver than those in DLY pigs.

### 3.2. Effects of the Plateau Environment on Antioxidant Activity in TPs and DLY Pigs

**Figure 2:**
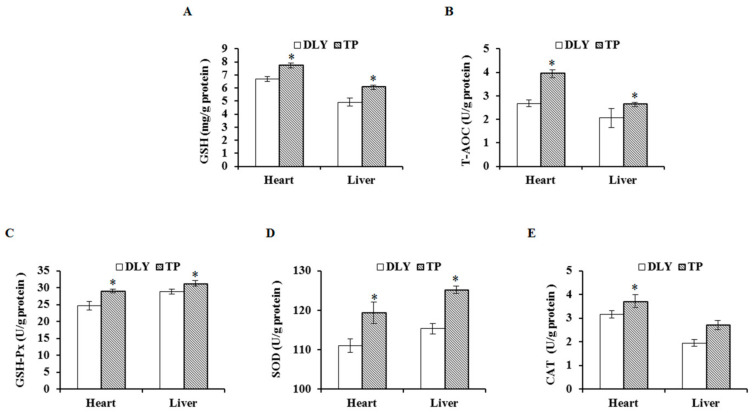
Effects of the plateau environment on GSH (**A**), T-AOC (**B**), GSH-Px (**C**), SOD (**D**), and CAT (**E**) in heart and liver of TPs and DLY pigs (*n* = 12). * Significant difference (*p* < 0.05). GSH = glutathione; T-AOC = total antioxidant capacity; GSH-Px = glutathione peroxidase; SOD = superoxide dismutase; CAT = catalase.

The effects of the plateau environment on antioxidant activity of TPs and DLY pigs are shown in Figure 2. Exposed to the plateau environment, TPs showed higher (*p* < 0.05) GSH, SOD, GSH-Px, and T-AOC activities in the heart and liver than those of DLY pigs, and higher (*p* < 0.05) CAT activity was only observed in the heart.

### 3.3. Effects of the Plateau Environment on mRNA levels of SOD, GSH-Px, and CAT in TPs and DLY Pigs

**Figure 3:**
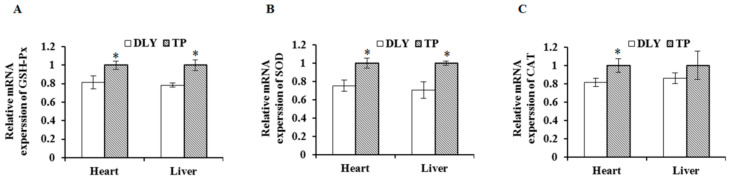
Effects of the plateau environment on mRNA levels of SOD (**A**), GSH-Px (**B**), and CAT (**C**) in heart and liver of TPs and DLY pigs (*n* = 12). * Significant difference (*p* < 0.05). SOD = superoxide dismutase; GSH-Px = glutathione peroxidase; CAT = catalase.

The effects of the plateau environment on mRNA levels of SOD, GSH-Px, and CAT in TPs and DLY pigs are shown in Figure 3. Exposed to the plateau environment, TPs showed higher (*p* < 0.05) mRNA levels of SOD, GSH-Px, and CAT in the heart and higher (*p* < 0.05) mRNA levels of SOD and GSH-Px in the liver than those of DLY pigs.

### 3.4. Effects of the Plateau Environment on mRNA and Protein Expression Levels of AMPK in TPs and DLY Pigs

**Figure 4:**
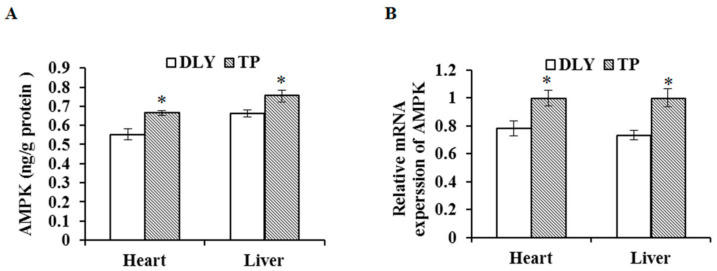
Effects of the plateau environment on protein (**A**) and mRNA (**B**) expression levels of AMPK in heart and liver of TPs and DLY pigs (*n* = 12). * Significant difference (*p* < 0.05). AMPK = adenosine 5′-monophosphate-activated protein kinase.

The effects of the plateau environment on mRNA and protein expression levels of AMPK in TPs and DLY pigs are shown in Figure 4. Exposed to the plateau environment, TPs showed higher (*p* < 0.05) mRNA and protein levels of AMPK in the liver and heart compared with those of DLY pigs.

### 3.5. Effects of the Plateau Environment on mRNA and Protein Expression Levels of p38 MAPK in TPs and DLY Pigs

**Figure 5:**
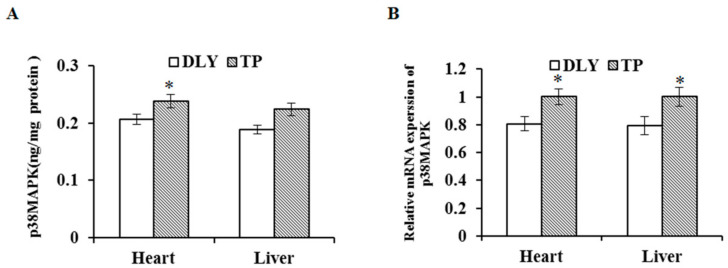
Effects of the plateau environment on protein (**A**) and mRNA (**B**) expression levels of p38 MAPK in heart and liver of TPs and DLY pigs (*n* = 12). * Significant difference (*p* < 0.05). p38 MAPK = p38 mitogen-activated protein kinase.

The effects of the plateau environment on mRNA and protein expression levels of p38 MAPK in TPs and DLY pigs are shown in Figure 5. Exposed to the plateau environment, TPs showed higher (*p* < 0.05) mRNA and protein levels of p38 MAPK in the heart and higher mRNA levels of p38 MAPK in the liver compared with those of DLY pigs.

### 3.6. Effects of the Plateau Environment on mRNA and Protein Expression Levels of Nrf2 in TPs and DLY Pigs

**Figure 6:**
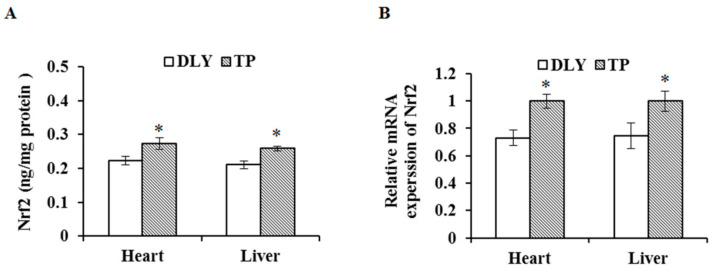
Effects of the plateau environment on protein (**A**) and mRNA (**B**) expression levels of Nrf2 in heart and liver of TPs and DLY pigs (*n* = 12). * Significant difference (*p* < 0.05). Nrf2 = nuclear factor erythroid 2-related factor 2.

The effects of the plateau environment on mRNA and protein expression levels of Nrf2 in TPs and DLY pigs are shown in Figure 6. Exposed to the plateau environment, TPs showed higher (*p* < 0.05) mRNA and protein levels of Nrf2 in the liver and heart compared with those of DLY pigs.

## 4. Discussion

Plateau environments can cause decreased oxygen, partial pressure, or oxygen content in circulating blood resulting in insufficient oxygen supply to tissues and cells and a series of stressed states [20,21,22]. Pigs are one of the most sensitive animals to hypoxic stimulation. Plateau hypoxia destroys tissue oxidative defense systems, enhances free radical levels, increases lipid peroxidation, and leads to tissue oxidative stress injury [4,23,24]. The biomarker levels of oxidative stress (tissue oxidation state) in animal cells and tissues significantly increase under hypoxia [25]. MDA is the free radical peroxidation reaction end product in cells and indirectly indicates the degree of cellular oxidative damage [26]. Diao et al. (2016) showed that the jejunal MDA content of TPs was lower than that of Yorkshire pigs [27]. Similarly, MDA levels were significantly lower in the heart and liver of TPs than that of DLY pigs in our study, indicating that the plateau environment caused less oxidative stress to TP organs than to DLY pig organs.

TPs are adapted to a life in the plateau areas, whereas DLY pigs live in low altitude areas; thus, there are major differences in oxidative stress and antioxidant defense responses and capacity between the two pigs. Cells form a complex antioxidant enzyme defense system, mainly composed of SOD, CAT, GSH-Px, and GSH, to protect the body from peroxidation damage [28,29,30]. SOD converts superoxide anion into hydrogen peroxide, which is then converted to harmless water by the catalytic activity of GSH-Px and CAT. CAT regulates hydrogen peroxide levels in vivo and protects Ryukyu proteins, while glutathione peroxidase oxidizes reduced glutathione in the presence of hydrogen peroxide, reducing the concentration of oxidized substances and relieving toxicity. During tissue hypoxia or ischemia, antioxidant enzyme activity decreases, while levels of oxygen free radicals and lipid peroxidation products increase [31,32]. Maimaitiyiming et al. (2014) suggested that the serum activities of SOD and GSH-Px are significantly higher, but the MDA level is significantly lower in rats living in the plateau than in low-altitude areas [33]. In the current study, the antioxidant indexes represented by CAT, SOD, and GSH activities in the heart and liver of TPs were significantly higher than those of DLY pigs, indicating that the antioxidant stress ability of TPs was higher than that of DLY pigs.

Hypoxic environments can cause an increase in reactive oxygen species and reactive nitrogen leading to oxidative stress, which affects the antioxidant defense system and activates key antioxidant signaling pathways [34,35]. Nrf2 activation under oxidative stress induces the transcription of genes encoding antioxidant enzymes such as NAD(P)H quinone dehydrogenase 1 (NQO1), heme oxygenase 1 (HO-1), SOD, CAT, GSH-Px, and GCL, which reduces the response to oxidative stress [36,37]. We found that TPs had higher Nrf2 protein and gene expression levels than those of DLY pigs in a plateau hypoxic environment, suggesting that the high TP antioxidant capacity in a plateau environment is closely related to the Nrf2 pathway.

AMPK is a common serine/threonine protein kinase in eukaryotic cells involved in cellular energy metabolism regulation; it is activated by oxidative stress and participates in antioxidant regulation [38,39]. After entering a plateau environment, the reduction in tissue oxygen may result in disordered energy metabolism and changes AMPK activity in skeletal muscle [40]. In this study, levels of AMPK protein and mRNA expression levels in the heart and liver of TPs were higher than those of DLY pigs. Hao et al. (2021) suggested that AMPK is important for antioxidant capacity of TP liver [41]. Furthermore, the activated AMPK is involved in the regulation of the Nrf2 signaling pathway [42]. The expression of antioxidant enzymes downstream of Nrf2 increased, while levels of reactive oxygen species decreased after AMPK activation [42].

The MAPK signaling pathway is involved in several biological processes, including regulating oxidative stress, inflammation, apoptosis, and cell proliferation [43,44]. p38 MAPK participates in a series of signaling pathways and responds to stress stimuli such as inflammatory cytokines and reactive oxygen species [45,46]. Activation of antioxidant gene transcription by Nrf2 is positively regulated by p38 MAPK [47]. In the current study, up-regulated expression of p38 MAPK and Nrf2 in the heart and liver of TPs could improve the expression levels of antioxidant proteins and genes. These above results show that activation of the p38 MAPK/Nrf2 pathway can reduce oxidative damage of the heart and the liver.

## 5. Conclusions

This study provides evidence that TPs have a stronger heart and liver antioxidant stress capacity than DLY pigs under a plateau hypoxic environment, resulting from AMPK/p38 MAPK/Nrf2-ARE antioxidant signaling pathways. These results provide insights into the relationship between the plateau hypoxic environment and oxidative stress, which maybe helpful in explaining the molecular mechanism of altitude response and adaptation in TPs. Furthermore, the approach (for example, dietary antioxidants) that enhanced the antioxidant capacity will improve pig production in the plateau environment.

## Figures and Tables

**Table 1:** Composition and nutrient levels of basal diets (%, as-fed basis).

Items	Content (%)
Corn	42.00
Soybean meal	11.50
Naked barley	13.00
Wheat bran	29.5
L-lysine hydrochloride	0.31
DL- Methionine	0.01
L-Threonine	0.03
CaHPO_4_•2H_2_O	0.85
NaCl	0.12
Limestone	1.68
Vitamin and mineral premix	1.00
Total	100.00
Nutrient levels	
Digestible energy, MJ/kg	11.70
Crude protein	14.01
Calcium	0.60
Phosphorus	0.40
Lysine	0.87
Methionine	0.24

**Table 2:** Primers used in the present study.

Gene	Forward Primer	Reverse Primer	Size (bp)	Accession No.
*SOD*	ATTCTGTGATCGCCCTCT	CTTTCTTCATTTCCACCTCT	100	NM_001190422.1
*GSH-Px*	TTGCCAAGTCCTTCTACGA	GAAGCCAAGAACCACCAG	188	NM_001115136.1
*CAT*	CGAAGGCGAAGGTGTT	CCACGAGGGTCACGAA	109	NM_214301.2
*AMPK*	TTGACTCGGCCCCATCCT	GTATGGCGTGCCCTTGGA	65	NM_001167633.1
*p38 MAPK*	ACAAGACAATCTGGGAGGTA	CACTGCAACACGTAACCC	116	XM_013977842.2
*Nrf2*	CACCACCTCAGGGTAATA	GCGGCTTGAATGTTTGTC	125	XM_021075133.1
*β-actin*	CTGCGGCATCCACGAAACT	AGGGCCGTGATCTCCTTCTG	147	DQ845171.1

## Data Availability

The data presented in this study are available from the corresponding author upon request.
